# Familial lecithin-cholesterol acyltransferase (LCAT) deficiency; a differential of proteinuria

**DOI:** 10.12860/jnp.2015.05

**Published:** 2015-01-01

**Authors:** Mohammed Mahdi Althaf, Hadeel Almana, Ahmed Abdelfadiel, Sadiq Mohammed Amer, Turki Omar Al-Hussain

**Affiliations:** ^1^Department of Medicine, Section of Nephrology, King Faisal Specialist Hospital and Research Center, Riyadh, Kingdom of Saudi Arabia; ^2^Department of Pathology and Laboratory Medicine, King Faisal Specialist Hospital and Research Center, Riyadh, Kingdom of Saudi Arabia; ^3^Department of Medicine, Dallah Hospital, Riyadh, Kingdom of Saudi Arabia

**Keywords:** Familial lecithin-cholesterol acyltransferase (LCAT) deficiency, Proteinuria, HDL-cholesterol

## Abstract

*Background:* Lecithin cholesterol acyltransferase (LCAT) is an important enzyme in cholesterol metabolism that is involved in the esterification of cholesterol. A lack of this enzyme results in deranged metabolic pathways that are not completely understood, resulting in abnormal deposition of lipids in several organs. Clinically, it manifests with proteinuria, dyslipidemia and corneal opacity with progressive chronic kidney disease resulting in end-stage renal disease.

*Case Presentation:* We herein present a case of a 30-year-old male with proteinuria that was not responsive to empiric management with angiotensin-converting enzyme (ACE) inhibitors and oral steroids. Physical examination revealed corneal ring opacity involving both eyes. Urinalysis revealed an active sediment. The 24-h proteinuria was 3.55 grams. Family history was positive for renal disease and dyslipidemia. Viral serology for human immunodeficiency virus (HIV), hepatitis C virus (HCV) and hepatitis B virus (HBV) were negative. Serum complements were normal and anti-nuclear antibody (ANA) was negative. We elected for a renal biopsy that revealed characteristic features of LCAT deficiency. The diagnosis of LCAT deficiency was established with a combination of clinical and pathological findings.

*Conclusions:* Currently renal prognosis is poor but conservative management with ACE inhibitors and lipid lowering therapy in addition to steroids has been shown to retard progression to end-stage renal disease. However newer therapies such as gene replacement and recombinant LCAT replacement are being studied with promising preliminary results.

**Implication for health policy/practice/research/medical education:**

Familial lecithin cholesterol acyltransferase (LCAT) deficiency is an autosomal recessive inherited disease that presents with corneal opacity, dyslipidemia and proteinuria with a poor renal prognosis where end-stage renal disease is common around the fourth decade of life. We present a case with classical presentation where diagnosis is confirmed by renal biopsy. Classical histological images are provided in this case report.

## 1. Introduction

In the metabolism of cholesterol, once free cholesterol is bound to HDL, cholesterol undergoes esterification to cholesterol esters by lecithin cholesterol acyltransferase (LCAT). In individuals with homozygous mutations in the LCAT gene, this enzymatic process is disrupted and results in extremely low serum levels of HDL-cholesterol. Clinically, it manifests with severe corneal opacities known as fish eye syndrome and the presence of target cells (1,2). We present a case where the patient presented with heavy proteinuria, obvious corneal opacities and a positive family history leading to the diagnosis of familial LCAT deficiency that was confirmed with renal biopsy.

## 2. Case Presentation

**Figure 1:**
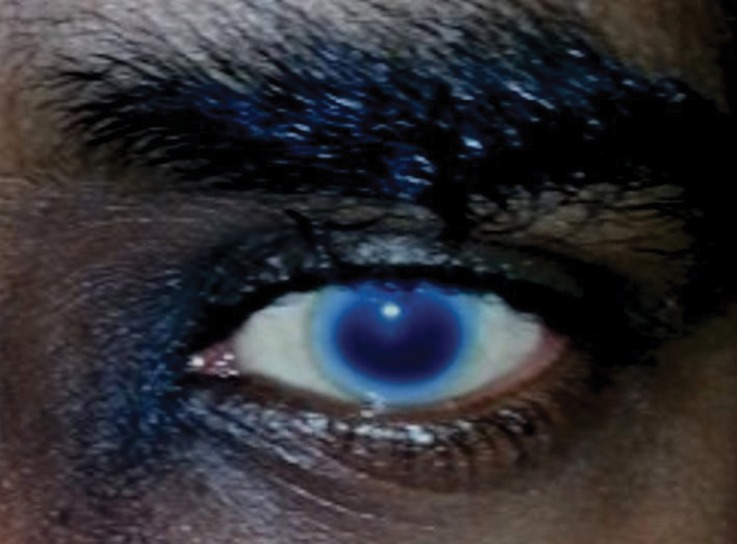
The most striking physical sign in our patient- corneal opacity.

A 30-year-old male was referred for persistent proteinuria from a community hospital. His past medical history was significant for hypertension and dyslipidemia. Treatment received was angiotensin-converting enzyme (ACE) inhibitors, statins and a trial of oral steroids without any change in baseline proteinuria. Family history was significant for familial dyslipidemia in several first degree relatives. His sister developed end-stage renal disease (ESRD) and was currently on hemodialysis; the cause of ESRD was not determined. The patient himself did not complain of any symptoms except for lower-limb edema on initial presentation. On examination, his blood pressure was 159/95 mmHg, pulse 82/min and was afebrile. He denied urinary symptoms, loin pain or analgesic use. Physical examination revealed a healthy looking young male of average built with body mass index of 24.8 kg/m^2^. Cardiovascular, chest, neurological and abdominal examination were unremarkable. The only striking finding was the presence of bilateral corneal ring opacities shown in Figure 1. Serum creatinine was 1.31 mg/dl, and serum electrolytes were within normal limits. Hemoglobin was 13 g/dl and platelets 200 × 10^9^/L. Serology for hepatitis B virus, hepatitis C virus, HIV 1 and 2 antibodies were negative. Serum total cholesterol was 309 mg/dl, triglycerides 545 mg/dl, LDL 177 mg/dl and HDL of 23 mg/dl. Liver enzymes and coagulation profile were within normal limits. Anti-nuclear antibody (ANA) was negative. Complement C3 and C4 levels were normal. Urinalysis showed protein 3+ and red blood cells 20-30 per high power field. The 24-h proteinuria was 3.55 grams. Renal ultrasound revealed bilateral echogenic kidneys with right renal length of 10.1 cm and left measuring 10.4 cm. Given the clinical scenario, we elected for renal biopsy after the patient was consented in order to establish a definitive diagnosis and identify any potentially reversible entity. Light microscopy with periodic acid-Schiff stain revealed glomerular mesangial matrix expansion and irregular thickening of the glomerular basement membranes as seen in Figure 2a. When stained with Jones Methenamine Silver ( Figure 2b ), it further illustrated the glomerular basement membranes with an irregular vacuolated appearance. These findings mimic the changes seen in stage 3 membranous glomerulopathy. Immunofluorescence microscopy was negative for immunoglobulins and complement components. Electron microscopy (Figure 3a, 3b, 3C) revealed lipid deposits appearing as abundant lacunae. The lacunae contains electron-lucent deposits with an electron-dense core involving the mesangial matrix as well as the glomerular basement membranes. These findings are in line with LCAT deficiency.

Figure 2Three perspectives on My Dog (a) Renal biopsy showing a glomerulus with mesangial expansion and irregular thickening of the glomerular basement membrane (GBM) (PAS stain, 400X). (b) GBM with an irregular vacuolated appearance mimicking the changes seen in stage 3 membranous glomerulopathy. (JMS stain, 400X)

**(a):**
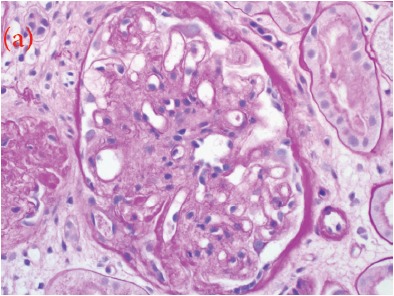

**(b):**
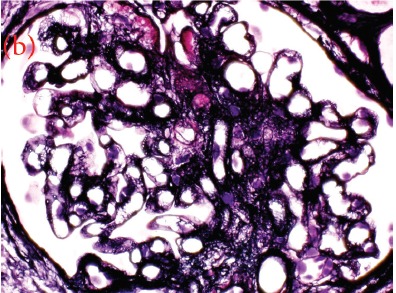

Figure 3(a) (b) and (c) Lipid deposits appear as abundant lacunae containing electron-lucent deposits with an electron-dense core involving the mesangial matrix and the glomerular basement membranes (electron micrograph)

**(A):**
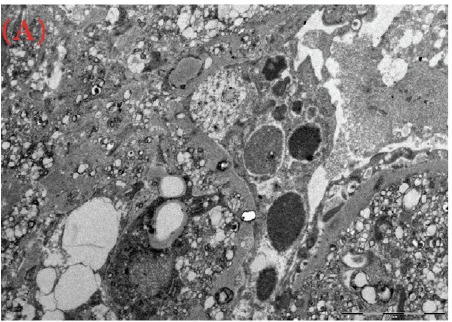

**(B):**
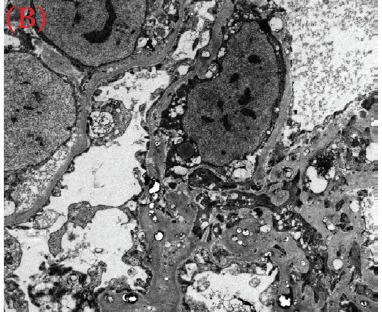

**(C):**
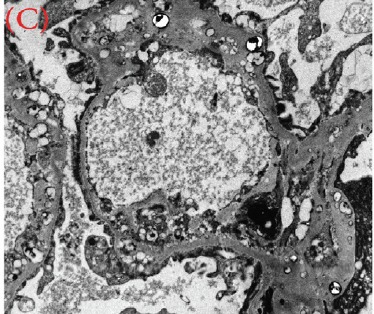

## 3. Discussion

The diagnosis of familial LCAT deficiency as the cause of proteinuria was then established in our patient given the positive family history and clinical information in conjunction with classical renal histopathological findings. Phosphatidylcholine-sterol O-acyltransferase is another name for LCAT; an enzyme bound to both HDL and LDL in plasma. This enzyme is responsible for the conversion of free cholesterol into cholesterol ester which renders it more hydrophobic (3). This product is then bound into the core of a lipoprotein particle that results in keeping the newly synthesized HDL spherical and maintaining the reaction to remain unidirectional as the particles are removed from the surface (4). The deficiency of LCAT results in alterations in the serum concentrations of unesterified cholesterol and lecithin eventually leading to abnormalities in the structure and composition of HDL and LDL particles (5). There are two forms of the disease, both of which are inherited in an autosomal recessive manner caused by mutations of the LCAT gene on chromosome 16q22. The first form is called ‘familial LCAT deficiency’ which is characterized by complete LCAT deficiency. The second form is known as ‘fish eye’ disease in which there is a partial deficiency. Both forms of the disease are extremely rare with just over 30 families together accounting for around 60 patients with familial LCAT deficiency. And a further 20 patients with fish eye disease have been reported in literature (4). The pathogenesis of renal injury in LCAT deficiency has not yet been fully elucidated. It has been suggested that LDL particles trapped in capillary loops may trigger endothelial and vascular injury (6). Renal lesions begin with lipid deposition in the glomerular basement membrane, mesangial expansion and lipid accumulation in the capillary sub-endothelium. Lipid deposits also occur in liver, spleen and bone marrow (7). The progression of renal disease is variable; these patients have progressive proteinuria and renal impairment leading to ESRD by the fourth decade of life (8,9). Currently there is no causal treatment for familial LCAT deficiency; lipid lowering therapy in addition to antihypertensive medication thus remains the mainstay of management. This combination has proven to retard the progression of the disease (10). The potential benefits of corticosteroid treatment have also been described (11). In those who develop ESRD; in addition to dialytic therapies, transplantation remains a viable option despite evidence of recurrence of the disease in renal allografts. Acceptable long-term results have been reported in transplant recipients with familial LCAT deficiency (12). Potential therapy with LCAT gene replacement and enzyme replacement are being explored. In one study, LCAT activity in plasma was restored using autologous adipocytes transfected with human LCAT via a retroviral vector (13). Recombinant human LCAT (ACP-501) demonstrated favorable results in LCAT knockout mice restoring LCAT activity, cholesterol efflux and lipid profiles. Recombinant human LCAT evaluated in familial LCAT deficiency patients in a phase 1 trial showed positive results and has given way to the ongoing clinical development of ACP-501 (14).

Currently, our patient is being treated with lipid lowering therapy in addition to ACE inhibitors. We look forward to the promising results from the phase 1 Recombinant human LCAT (ACP-501) study and eagerly await for the final product that can change the future of all patients with LCAT deficiency.

## 4. Conclusions

Familial LCAT deficiency is an autosomal recessive disorder that inevitably progresses to ESRD. This case illustrates the utility of a renal biopsy in determining the cause of proteinuria.

## Authors’ contributions

All authors wrote the paper equally.

## Conflict of interests

None of the contributing authors have any conflict of interest, including specific financial interests or relationships and affiliations relevant to the subject matter or materials discussed in the manuscript.

## Funding/Support

None.
